# Assisted or Hastened Death: The Healthcare Practitioner’s Dilemma

**DOI:** 10.5539/gjhs.v4n6p87

**Published:** 2012-08-30

**Authors:** Rod D MacLeod, Donna M Wilson, Phillipa Malpas

**Affiliations:** 1 Hammond Care, Greenwich Hospital, Sydney, NSW, Australia; 2 Northern Clinical School, University of Sydney, Australia; 3 Faculty of Nursing, University of Alberta, Edmonton, AB Canada; 4 Department of Psychological Medicine, University of Auckland, New Zealand

**Keywords:** hastened death, euthanasia, palliative care, ethics, assisted suicide, physician assisted suicide

## Abstract

Assisting or hastening death is a dilemma with many ethical as well as practical issues facing healthcare practitioners in most countries worldwide now. Various arguments for and against assisted dying have been made over time but the call from the public for the legalisation of euthanasia and assisted suicide has never been stronger. While some studies have documented the reluctance of medical and other healthcare professionals to be involved in the practice of assisted dying or euthanasia, there is still much open debate in the public domain. Those who have the most experience of palliative care are strongest in their opposition to hastening death. This paper explores salient practical and ethical considerations for healthcare practitioners associated with assisting death, including a focus on examining the concepts of autonomy for patients and healthcare practitioners. The role of the healthcare practitioner has clearly and undoubtedly changed over time with advances in healthcare practices but the duty of care has not changed. The dilemmas for healthcare practitioners thus who have competent patients requesting hastened death extends far beyond acting within a country’s laws as they go to the very heart of the relationship between the practitioner and patient.

*“I will neither give a deadly drug to anybody if asked for it, nor will I make a suggestion to this effect”* Hippocrates (5^th^ Century BC)

## 1. Introduction

Public debate over legalizing assisted or hastened death is occurring in many countries including the UK, Australia, New Zealand, Canada and the United States where there is understandably much academic and healthcare professional interest as well. This interest is not surprising, as although much historical and current opposition to hastened death exists, there is a growing list of countries where assisted suicide and/or euthanasia take place now under legal sanction; these include the Netherlands, Belgium, Switzerland, Luxembourg and Oregon, Montana and Washington in the United States. Many arguments for and against assisted suicide and euthanasia have been made over the years. Most of these have not considered what it would be like for the healthcare practitioner to be involved in the decision to take a life and also involved in the practice of actually taking a life. The focus in this paper is on the practical as well as personal ethical or moral considerations of assisted suicide and euthanasia for healthcare practitioners. To this end, a history of this debate is outlined. Throughout this paper, major dilemmas are identified, with these included to aid in the discussion of whether assisted or directly hastened death is something that healthcare professionals (most often physicians) should be responsible for. Various definitions for assisted suicide have been proposed over the years (see appended note), but for the purpose of this article we draw on the European Association of Palliative Care (EAPC) ethics task force’s definition: “intentionally helping a person commit suicide by providing drugs for self-administration, at that person’s voluntary and competent request” (p. 98). Euthanasia was defined as “intentionally killing a person by the administration of drugs, at that person’s voluntary and competent request” (Materstvedt, 2003, p. 98). This paper starts with a historical background to the current discussions concerning assisted dying and euthanasia. Literature from general medical, bioethical, legal, and palliative care journals is included to illustrate contemporary considerations of the views of healthcare practitioners. Practical and ethical considerations in this area are also explored. Finally, as much has been written about the significance of respect for autonomy and the healthcare professional’s duty of care – both concepts are summarized before conclusions are drawn.

## 2. History

The issue of assisting or directly hastening a patient’s death is not a contemporary dilemma. In 5th century BC, Hippocrates explicitly stated that new physicians must refrain from such a practice, swearing an oath before the gods of healing that they will uphold ethical and professional standards to preserve life whenever possible (Edelstein, Temkin & Temkin, 1967). It is also clear from ancient scholars that a self-administered death was not explicitly prohibited, and furthermore, that some physicians were instrumental in helping terminally-ill or fatally injured individuals to die (Gillon, 1969; Stolberg, 2007).

The emergence of the Judeo-Christian era (13^th^ Century) brought about a major change in social values on life and death, and effectively put an end to the idea of suicide or assisted suicide being permissible (Steele & Hill, 1976). St Thomas Aquinas has been identified among others as being influential in proposing an orthodox Christian view of suicide violating one’s duty both to self and to God, and it could therefore never be justified in any form. Regardless, healthcare practitioner opinion on the permissibility of assisted suicide and euthanasia remained divided at the beginning of the 20^th^ century. One 1899 editorial in the journal The Lancet, for instance, argued that “the question of painlessly shortening the existence of a fellow-being are hardly to be taken seriously” (Anonymous, 1899). The editor’s opinion was based on the potential for abuse “by the unscrupulous persons who are unfortunately to be found in every rank of life [that], the medical profession would never countenance it” (p. 532). Twenty years later, other authors also questioned the practical application of any law that would allow assisted suicide or euthanasia, but they did acknowledge that if the law could “define and recognise a “hopeless” case”, then “this function (of assisted suicide or euthanasia) must be entrusted to the medical profession” (Anonymous, 1919, p. 803). The fear then as now was that if assisted suicide and/or euthanasia were practiced openly, it would be abused if lay persons could practice it upon others.

However, there is little doubt that throughout human history those charged with providing healthcare services have assisted very-ill individuals to die more rapidly than nature would have allowed. In 1924, Chandler – under the title, Cocaine for Euthanasia – wrote that “For several years I have used cocaine in the last stages of phthisis” (Chandler, 1924, p. 629). While he doesn’t explicitly admit to deliberately and actively ending any terminal patient’s life, the title of his letter is illustrative of this intent as well as being instructive as to the method he used for terminating life. Much less information is available on the historic views of other healthcare professionals, such as nurses and pharmacists. There has also been periodic but on-going debate about legalizing or permitting assisted suicide and/or euthanasia for more than 20 years in many parts of Europe, North America, and Australasia. However, never has the call to formally debate these issues been as vocal and organised as it is today. Much of this current and historic debate has been focused on suffering at the end of life. In 1961, an editorial in The Lancet identified the “gross inadequacy of our provision for decent, seemly quitting of life, with relievable pain and distress relieved and comfort given” (Anonymous, 1961, p. 351).

## 3. Current Considerations

Thankfully, the tremendous advances in health care over the past five decades have benefited society in numerous diverse ways; perhaps the most significant of these is the ability to sustain and prolong life where once individuals would simply have died. Although much scientific and healthcare progress is still not a reality for many of the world’s poorer populations, ill persons today can and do live much longer in most countries (Macleod, 2012). These advances have also meant that it is increasingly possible for people to have long terminal illnesses and long dying processes. Death thus remains a reality for all people, with the specialty of palliative care having proliferated globally since the 1970s as a way to prevent and address end-of-life suffering. Palliative care is an active form of care that focuses on improving quality of life and it aims to neither hasten nor postpone death, and this focus is made clear to recipients of such care. Regardless, some terminally-ill and some dying people request assisted suicide or euthanasia as they would trade quantity of life for quality of life, or at least a certainty to the end of life. The dilemma for healthcare practitioners then is how to balance the patient’s view of quality versus quantity of life against the historical and culturally developed roles and responsibilities of their healthcare profession to support life and prevent suffering.

In New Zealand, there is evidence through two population-based surveys that the majority of those surveyed support assisted suicide and/or euthanasia (Gendall, 2003; Voluntary Euthanasia Society, 2008). Internationally, studies from other various population groups also conclude consistent support for the right to hastened death, often with physicians named as the agents who would be responsible for assessing and enabling assisted suicide or euthanasia (Emanuel, Fairclough & Emanuel, 2000; Chapple, Ziebland, McPherson & Herxheimer, 2006; Wilson et al., in press).

Within the medical profession, support for physician-assisted suicide (PAS) and/or euthanasia is evident among a relatively small proportion, with the level of support depending on the survey timing and also the country of survey (Bachman et al., 1996; Cohen, Fihn, Boyko, Jonsen & Wood, 1994; Neil, Coady, Thompson, & Kuhse, 2007). For instance, Seale (2009) undertook a survey of 3,733 medical practitioners in the UK, and found the majority view was not in support of PAS, a view that was at odds with the general public’s more favorable view of it. Among the physicians surveyed, palliative specialists were particularly opposed. A recent systematic literature review of UK physician attitudes concerning PAS and euthanasia similarly indicated that UK doctors generally opposed the introduction of both practices (McCormack, Clifford, & Conroy, 2012). Religious affiliation is one of the factors identified to date as influencing a physician’s stance on assisted suicide and/or euthanasia (Christakis & Asch, 1995; Crane, 1977; Curlin, Nwodim, Vance, Chin & Lantos, 2008; Finlay, 1985; Seale, 2009). Other studies have found additional select factors were associated with physician willingness or unwillingness to participate in hastening death such as their specialty, gender, and ethnic group (Cohen et al., 2008; Heath, 2012; Meier et al., 1998).

Another major issue, beyond the question of whether death hastening can or should be legally performed, is who will be responsible for the decisions about which persons can or cannot be assisted to die. Clearly, palliative care physicians are the least likely to want to be involved in these decisions and yet arguably these are the most relevant physicians to be involved. A related concern is based on the person or persons who will be involved in the actual practice of hastening death. It would seem reasonable that healthcare practitioners would be instrumental in ensuring that assisted suicide and euthanasia are conducted properly when it has been authorized. However, despite public opinion generally favoring PAS, physicians are rarely supportive of the view that they be the instrument through which death hastening should occur. Other healthcare professions have also expressed a similar sentiment. For instance, one study that explored the influences of personal and professional attributes of physicians, nurses, and social workers on the willingness to endorse assisted suicide, noted that, “the respondents at the hospital devoted to the care of the terminally ill, were significantly less willing to endorse assisted suicide than [those at the other sites]” (Portenoy et al., 1997, p. 282).

Despite the fact that PAS and euthanasia have only been legally available to Dutch citizens since 2002, both have been tolerated within Dutch society for several decades (Griffiths, Weyers & Adams, 2008). Within Europe, Switzerland, Belgium and Luxembourg currently allow PAS. Switzerland is the only country in the world where the act of assisted dying can be conducted by someone who is not a physician. Furthermore, non-Swiss persons are permitted to take advantage of the Swiss law, with individuals travelling to Switzerland to die by assisted means. Oregon is another interesting jurisdiction, as in this US state since 1997, a terminally-ill person who has been diagnosed as having less than six months left of life may request and receive a prescription from a physician or nurse practitioner and they are then at liberty to take or not take the medication to end their life. In these Oregonian cases, the ill person may be assisted by a family member or home care nurse or anyone else to commit suicide. If the person becomes too advanced in their illness to pick up and/or swallow the medication, the pills could be crushed and introduced via a feeding tube or other means by their caregivers. Within the US, several other states also allow assisted suicide. Washington (2009) allows assisted suicide under a *Death With Dignity* (DWD) law. In 2009, the Supreme Court in the state of Montana also clarified the law’s position that neither state law nor public policy prevented the prescribing of lethal drugs to terminally-ill patients who want to end their lives (O’Reilly, 2010). Several other states are currently considering their own DWD laws (i. e. Vermont, Massachusetts New York, Pennsylvania, and Hawaii). Similarly, in Canada, a Supreme Court judge ruled in mid-2012 that persons who cannot commit suicide themselves are disadvantaged by the current law that prevents assisted death practices from occurring openly (Wilson et al., in press). Quebec, a Canadian province, has also studied the matter of assisted death and completed a report in late 2011 that recommends both assisted suicide and euthanasia should be practiced in the province for those who wish it, and also that palliative care should be more accessible and available to those who need it (Wilson et al.). Despite these legal developments, requests for assisted suicide and euthanasia raise a number of challenging practical and ethical considerations for healthcare practitioners. It is to these we now turn.

## 4. Healthcare Practitioners: Practical Considerations

Assessing capacity to decide on any intervention is a key component of this discussion, however, surprisingly little commentary is made on this issue in the literature. Mental incapacity is common in acutely-ill hospital and nursing home patients, and yet it has been suggested that clinicians tend not to recognise incapacity (Sessums, Zembruka & Jackson, 2011). Screening methods for cognitive impairment are needed for detecting those with or without mental capacity, to ensure that requests for assisted suicide or euthanasia are made by competent persons. There seems little agreement on the most appropriate assessment tool(s) to be utilised, however (Sessums, Zembruka & Jackson). Furthermore, it is not evident which tool(s) for recording personal requests for assisted suicide or euthanasia are the most appropriate, as these need to be completed before incompetence occurs with advancing ill health. Advance directives are one tool in which the wishes of persons may be clearly recorded.

Depression is another major consideration, as patients having both cancer and depression experience more physical symptoms, have poorer quality of life, and are more likely to have suicidal thoughts or a desire for hastened death than cancer patients who are not depressed (Breitbart et al., 2000). The relatively common end-of-life symptoms of hopelessness, loss of meaning, and existential distress have been proposed as the core features of the diagnostic category of demoralization syndrome (Kissane, Clarke & Street, 2001). Kissane and colleagues differentiate this syndrome from depression and assert that demoralization is associated with chronic medical illness, disability, bodily disfigurement, fear of loss of dignity, and social isolation. Because of the sense of impotence or helplessness with impending death and terminal illnesses, those with the syndrome may progress to express a desire to die or to commit suicide. Similarly, those persons who are depressed and feel hopeless have been found to have an increased desire for death (Breitbart et al., 2000; Hendon & Foley, 2008; Mehnert, Vehling, Höcker, Lehmann, & Koch, 2011).

Another issue is the direct personal impact (emotionally, professionally, spirituality and psychologically) on the doctors and other healthcare practitioners who have a socially and professionally-endorsed mandate to prevent premature death and actively address suffering but who may be asked to assist patients to die. The impact of this request can be profound and long-lasting. In countries where assisted suicide is illegal, doctors who do assist their patients to die may be entirely unable to access help for any personal issues that may arise for them as a consequence of their actions. Patients who request help to die from their doctor are also vulnerable as this help must occur in secret, and yet practitioners are extremely vulnerable to being discovered and reported if someone witnesses the death or becomes aware of it and discloses it to the authorities. As the death takes place covertly, practitioners cannot safely ‘share the load’ with colleagues to get emotional and professional support. Macleod (2012) also suggests another pressure that doctors may become weighed down by the psychological issues associated with their patients who are dying. No doubt, physicians and other healthcare professionals may find themselves in the position of being begged at times by patients or their family members to end a life. This burden to assist or hasten dying comes with a cost. Large proportions of Dutch doctors, for instance, have expressed feelings of discomfort following euthanasia or assisted suicide (Georges, The, Onwuteaka-Philipsen & van der Wal, 2008; Haverkate, van der Heide, Onwutekea-Philipsen, van der Maas & van der Wal, 2001; van Marwijk, Haverkate, van Royen, & The, 2007). A US study also found 24% of physicians regretted being involved, with 16% reporting that the emotional burden of having performed euthanasia or PAS had adversely affected their medical practice (Emanuel, Daniles, Fairclough, & Clarridge, 1998). Some Flemish GPs in Belgium have also found the practice of performing euthanasia to be difficult (Sercu et al., 2012).

Concern that PAS and/or euthanasia will result in negative repercussions for hospice and palliative care (for instance, through a reduction in palliative services funding/support) presents another significant dilemma. Most national hospice/palliative organisations have position statements indicating opposition to euthanasia and assisted suicide. However, some argue that there are no negative repercussions in having both palliative care and PAS available for dying patients. Bernheim and colleagues (2008) would support this view as they reflected on “the effect of the process of legalisation of euthanasia on palliative care by reviewing published historical, regulatory, and epidemiological evidence in Belgium” (p. 864) and stated, “we found few professional stances contending that palliative care and legalisation of euthanasia are antagonistic, no slippery slope effects, and no evidence for the concern of the European Association for Palliative Care that the drive to legalise euthanasia would interfere with the development of palliative care. Rather, there were many indications of reciprocity and synergistic evolution” (p. 866).

Another dilemma is the use of sedation near the end of life – at times used as a strategy of last resort to minimise distressing refractory symptoms that are difficult to manage - not hasten death, but with this practice resulting in confusion about PAS. There is now good evidence to support the view that the dose of morphine or other analgesics used in sedation near the end of life and the rate of dose increase has no effect on the time of death. Recent studies have concluded that palliative sedation therapy does not hasten death when used to manage refractory symptoms (Maltoni et al., 2009; Rady & Verheijde, 2010). However, clear communication to explain the purpose of such sedation is needed, as an increase in the use of strong opioids near the end of life can cause confusion about death hastening in the minds of healthcare staff and families. Trust in the healthcare team is one of the most important aspects for quality end-of-life care (Heyland et al., 2006), and families and patients need to be able to trust in their clinicians. Similarly, effective and empathetic communication is essential to avert differences in understanding about the goal of all end-of-life medications and treatments (Steinhauser et al., 2000).

Other factors also need to be considered, such as those in the area of quality of life for dying persons (Singer, Martin & Kelner, 1999). Together, these factors highlight the importance or relevance of providing effective palliative care (Ganzini et al., 2000). Effective interventions for pain and psychological distress could produce a change in view among persons who are seeking an early end to a difficult life.

## 5. Healthcare Practitioners: Ethical Considerations

While laws that permit or prohibit assistance to hasten death are evident in most jurisdictions around the world, the ethical dimensions associated with them remain deeply contentious. This is not surprising, given that the death of any individual is neither trivial nor inconsequential for those involved. Assisting an individual to die, with or without consent and regardless of the circumstances, is unethical for some because of the inherent value and sanctity of all human life (Kass, 1991). For others, concerns about the validity and authenticity of a patient’s consent to request assistance to die, the potential for widespread abuse of society’s most vulnerable persons, and disagreement about the need for assisted dying in light of other avenues such as palliative or hospice care, raise resistance among some healthcare professionals to both the concept and practices of assisted death (Hendon & Foley, 2008; Pereira, 2011). Yet some commentators indicate assistance to hasten death is ethically permissible because competent individuals have a right to request and receive assisted death (although often in qualified circumstances) (Bartels & Otlowski, 2010). This stance would imply that denying terminally-ill individuals an assisted death who wish it is perhaps cruel and unfair. In these cases, they could argue that an assisted death may be more humane than allowing nature to take its course (Rachels, 1974). Healthcare practitioners confronted with a patient’s request for assisted suicide or euthanasia may thus encounter a tension between balancing the self-determined interests of the patient with their own autonomy, and the broader socially accepted duty of care by practitioners to patients at the end of life.

## 6. Respect for Autonomy: Patients and Healthcare Practitioners

The past several decades have seen the principle of respect for patient autonomy assume a central place in health care. Indeed, respect for the self-determining decisions or interests of patients now underpins most professional codes of practice and also patient codes of rights (Chisholm & Askham, 2006; GMC, 2006; Health and Disability Commissioner, 2009). The dilemma now is that fear of losing autonomy or dignity during the dying process could lead some patients to request a hastened death. In a study investigating physician attitudes and behaviours about (hypothetical) end-of-life decisions that hastened death, researchers found physicians who would acquiesce to a patient’s request for an assisted death stated that respect for the patient’s autonomy was important in their decision-making (Fried, Stein, O’Sullivan, Brock, & Novack, 1993). Physicians who would not comply with a patient’s request for an assisted death agreed that respect for autonomy was significant but instead citing other factors such as ethical and legal concerns (Fried et al., 1993).

Evidence from Oregon’s first year of legalised PAS is also of relevance here, as this shows many terminally-ill patients worried about their loss of autonomy and loss of bodily functions, leading the authors to suggest that controlling the time of death was important to them (Chin, Hedberg, Higginson & Fleming, 1999). The authors further concluded that “the decision to request and use a prescription for lethal medications during the first year of legalized physician-assisted suicide in Oregon was associated with views on autonomy and control, not with fear of intractable pain or concern about financial loss” (Chin et al., p. 582). Similar findings were evident in the second year of legislation in Oregon (Sullivan, Hedberg & Fleming, 2000) and after (Ganzini, Dobscha, Heintz & Press, 2003). In addition, French researchers who were investigating requests to hasten death in palliative care settings also found loss of autonomy was a factor for patient decision-making (Ferrand et al., 2012). Yet Salem (1999) argued that PAS, far from respecting an individual’s autonomy, is in fact an impediment to it. Salem claimed that PAS ultimately extends the power of physicians in regulating death. In responding to Bartels and Otlowski’s (2010) claim that respect for individual autonomy underpins a right to die (while recognising that such a right is not absolute), Prichard (2012) objected by stating that the importance attached to such a right is overstated. Prichard and others thus claim that our right to do with our bodies what we will is relevantly different to a right to demand hastened-death assistance from others. However, even where a right to die is recognised in a country, it does not oblige someone to assist another person to die; autonomy’s reach is thus limited (Putnam, 2009).

Some feminist scholars have raised other considerations of relevance claiming that autonomy - an individualistic notion that focuses on separateness - fails to adequately account for the complex web of social relationships that underpin families, friendships, and relationships with colleagues (Donchin, 2000; Heath, 2012; Parks, 2000). This thick relationship is especially relevant within the context of assisted suicide/euthanasia where family dynamics may influence and steer decision-making at the end of life. Wolf (1996) suggested gender thus deserves considerable analysis in the debate over assisted suicide and euthanasia, noting that “the long history valorising women’s self-sacrifice may be expressed in women’s requesting assisted suicide or euthanasia, leading to her assertion that “we had better determine whether tacit assumptions about gender are influencing the enthusiasm for legalisation” (p. 285). In other words, how women come to make decisions about assisted suicide/euthanasia may require deeper analysis to ensure that a decision is truly autonomous and free from coercive influences. There is also an inherent paradox in acquiescing to a patient’s request to assist their death (on the grounds that doing so respects their autonomy), as the very act of hastening death effectively ends one’s autonomy - once and for all.

Clearly, autonomy arguments must also consider the healthcare practitioner as an autonomous agent. Some have argued that respecting healthcare professional autonomy necessitates that they cannot be required – against their will - to assist patients to die, an argument that obliges some practitioners to refer patients to someone else who will help them achieve a hastened death. To this end, laws in some countries have been crafted so that the autonomy of practitioners is not compromised in jurisdictions that allow assisted suicide and euthanasia, even when they are also crafted to protect the death-hastening decisions of patients. The Netherlands and Belgium illustrate this situation.

## 7. Duty of Care and the Role of the Healthcare Practitioner

There is little doubt that the roles of healthcare practitioners have changed as health care has advanced. As technologies, treatments, and care have improved over time, so too has the efficacy of healthcare practitioners. This advancement can be clearly illustrated in the way that palliative care has developed in many parts of the world. With improvements in the medications used to manage refractory end-of-life symptoms, and a greater awareness of how individuals and their families cope with the dying process, palliative care has evolved to shift the focus of care away from cure when cure it no longer an option. Palliative care has also become most successful in addressing the spiritual, emotional, physical, and social needs of patients and their families at the end of life. Thus the role of healthcare providers near the end of life has shifted over time from that of healer and decision-maker to one that may be better articulated as a chaperone or companion through a highly normal but often difficult life process. Drawing closer to the patient (and family) who is near the end of life is a central aspect of the palliative care approach, as it underpins a crucial aspect of the duty of care for palliative care providers.

Niall Dickson, the Chief Executive of the General Medical Council (UK), recently pointed out: “the issue of assisted suicide is complex and sensitive. We already have clear guidance for doctors that they must always act within the law and assisting or encouraging suicide remains a criminal offence. This guidance will not in any way change the legal position for doctors. It is not our role to take a position on whether or not the law should be changed; that is a matter for the relevant legislature” (General Medical Council, 2011). The view that a doctor’s role may legitimately include assisting patients to die in some circumstances strikes some as placing “the very soul of medicine on trial” (Gaylin, Kass, Pellegrino & Siegler, 1988, p. 2139). Others would appear to agree with this view (Kass, 1989; Paris, 2009; Pellegrino, 1992). For instance, Rosenblum and Forsythe (1990) claimed that were doctors to engage in assisted suicide or euthanasia: “the fundamental distinction between the physician as healer and the physician as killer would be vaporized: morality would be severed from mortality” (p. 25). This stance stems from the view that doctors have a duty in law to protect life and further the health of their patients. Similarly, others argue that doctors are educated to preserve life, not take it; their intent must be to provide care and not death (Foley, 1997). Randall and Downie (2010) thus claim it is irrational to consider assisted suicide and euthanasia as part of the doctor’s role. They argue that neither falls within the remit of treatments or healthcare interventions and so they cannot be in a patient’s best interests (Randall & Downie).

Yet, at the same time, other clinicians and academics have persuasively argued that the role of the doctor and other healthcare professionals may extend to assisting a competent patient to die when that person’s life has become unbearable for them (Gill, 2005; Quill, 1991; Quill & Battin, 2004; Wanzer et al., 1989). Wanzer and colleagues (1989) would support this role within the context of competent patients near the end of life, as their study found “all but two of us believe that it is not immoral for a physician to assist in the rational suicide of a terminally ill person” (p. 848). They also stated that this assistance must be a practice of last resort. Euthanasia and PAS, as practices of last resort, constitute a view also defended by others (Ganzini & Block, 2002; van Delden, 1999). Indeed, some physicians openly report that it is their duty to conduct assisted dying in some circumstances (Hussain & White, 2009); one general practice physician in that study stated: “If somebody did make that autonomous choice [for PAS], I wouldn’t wish to disengage myself from it and actually it is probably the last service you can render to somebody in that situation and there is something rather cowardly, in the absence of a moral objection, [about] leaving it to somebody else” (p. 846).

This dilemma about whether assisted or hastened death is within or beyond the role of healthcare practitioners is clearly very complex. It is perhaps instructive to consider what occurs in Belgium, where assisted dying measures such as PAS or euthanasia are said not to be related to a lower use of palliative care and they also often occur within the context of multidisciplinary care (van den Block et al., 2009). In addition, a number of legal judgments on withholding and withdrawing treatment, mainly in English courts, have shown that the courts do not consider that protecting life always takes precedence over death and related considerations. However, whether such judgments extend to healthcare practitioners actively assisting their patients to die is still a deeply contested view in many countries, including those where evidence shows assisted suicide and/or euthanasia are occurring illegally (Emanuel, Fairclough, Daniels & Clarridge, 1996; Kuhse, Singer, Baume, Clark & Rickard, 1997; Meier et al., 1998; Mitchell & Owens, 2004; Seale, 2006).

## 8. Conclusions

The many dilemmas for healthcare practitioners who have competent patients (and/or their families) requesting hastened death extends far beyond acting within their country’s laws for engaging in a sanctioned or unsanctioned practice. Even where the law is clear that an assisted death is permissible under certain circumstances, the practical and ethical issues that result from considering and acting upon a request are complex and troubling for many healthcare practitioners. While many patient rights’ codes and professional codes of practice elevate the autonomy and best interests of the patient, laws currently forbid assisting a patient to die early in all but a few jurisdictions. Regardless, hastened death requests are occurring in countries where hastened death is not sanctioned through law, and healthcare professionals in these countries have a greater personal burden as compared to healthcare professionals who can openly practice or not practice assisted suicide/euthanasia in countries where it is now legally permissible.

Within the medical and bioethical literature, there is support for, as well as considerable opposition to, the involvement of healthcare practitioners in assisted suicide and euthanasia. Among all healthcare providers, palliative care specialists appear to be the ones who most often reject hastened death as a practice they condone and are willing to be involved in. For many, allowing practitioners to hasten the death of a patient speaks more of abandonment at a time when patients (and their family) need to be drawn together for higher quality life until death. These differences among healthcare professionals must be understood and respected regardless of the current urgency from some to legalize hastened death.

## Appendix—Clarifying Terms:

In order to discuss the issues pertinent to assisted suicide and physician-assisted suicide specifically, it is important to define what is meant by these terms, and that of euthanasia. Several commentators have drawn attention to the confusion that often accompanies these terms (Mitchell, 1999; Sellman, 1995; Vamos, 2012; Silveira, DiPiero, Gerrity & Feudtner, 2000; Neil, Coady, Thompson & Kuhse, 2007) with some authors claiming that the term euthanasia is so loaded that it must not be used with regards to current end-of-life care (Michalson & Reinhart, 2006). Notably, use of the term “suicide” within physician-assisted suicide is also unsatisfactory for many who claim that assistance to die at the end of life precisely because it is the end of life, is relevantly different to the ordinary usage of suicide which is generally viewed as a tragedy because it ends a life that is not in a terminal or near death state. Furthermore, some have stated that the terms “physician-assisted” or “doctor-assisted” focus attention onto the clinician, even though they ought not to be the focus. By labeling the event “physician assisted dying,” we concentrate on the actions of the physician, almost making it sound as though the physician is a decision-maker, rather than orientating ourselves toward a discussion of the entire decision-making process and the dying person (Bender, 1991; Tucker & Steele, 2007). Many are now indicating that the term “hastened death” be used as it is a more accurate description (Breitbart et al., 2001; Goldney, 2012). This distinction about suicide and hastened death is poignantly illustrated by Ethan Remmel who – when he was dying of cancer - said it’s “not a choice between living and dying but between different ways of dying” (Remmel, 2011).

## References

[ref1] Anonymous (1989). Euthanasia. The Lancet.

[ref2] Anonymous (1919). Euthanasia. The Lancet.

[ref3] Anonymous (1961). Euthanasia. The Lancet.

[ref4] Bachman J. G, Alcser K. H, Doukas D. J, Lichtenstein R. L, Corning A. D, Brody H (1996). Attitudes of Michigan physicians and the public toward legalizing physician-assisted suicide and voluntary euthanasia. New England Journal of Medicine.

[ref5] Bartels L, Otlowski M. A (2010). Right to die? Euthanasia and the law in Australia. Journal of Law and Medicine.

[ref6] Bender L (1991-1992). Feminist analysis of physician-assisted dying and voluntary active euthanasia, a symposium: Recent work in feminist legal thought. Tennessee Law Review.

[ref7] Bernheim J, Deschepper R, Distelmans W, Mullie A, Bilsen J, Deliens J (2008). Development of palliative care and legalisation of euthanasia: Antagonism or synergy?. British Medical Journal.

[ref8] Breitbart W, Rosenfeld B, Pessin H, Kaim M, Funesti-Esch J, Galietta M, Brescia R (2000). Depression, hopelessness, and desire for hastened death in terminally ill patients with cancer. Journal of the American Medical Association.

[ref9] Breitbart W, Rosenfeld B, Pessin H, Kaim M, Funesti-Esch J, Galietta M, Brescia R (2000). Depression, hopelessness, and desire for hastened death in terminally ill patients with cancer. Journal of the American Medical Association.

[ref10] Chandler F. G (1924). Cocaine for euthanasia. The Lancet.

[ref11] Chapple A, Ziebland S, McPherson A, Herxheimer A (2006). What people close to death say about euthanasia and assisted suicide: A qualitative study. Journal of Medical Ethics.

[ref12] Chin A. E, Hedberg K, Higginson G. K, Fleming D. W (1999). Legalized physician-assisted suicide in Oregon--the first year’s experience. New England Journal of Medicine.

[ref13] Chisolm A, Askham J (2006). A review of professional codes and standards for doctors in the UK, USA and Canada.

[ref14] Cohen J. S, Fihn S. D, Boyko E. J, Jonsen A. R, Wood R. W (1994). Attitudes toward Assisted Suicide and Euthanasia among Physicians in Washington State. New England Journal of Medicine.

[ref15] Cohen J, van Delden J, Mortier F, Lofmark R, Norup M, Cartwright C, Bilsen J, on behalf of the Eureld Consortium (2008). Influence of physicians’ life stances on attitudes to end-of-life decisions and actual end-of-life decision-making in six countries. Journal of Medical Ethics.

[ref16] Christakis N. A, Asch D. A (1995). Physician characteristics associated with decisions to withdraw life support. American Journal of Public Health.

[ref17] Crane D (1977). The sanctity of social life: physicians’ treatment of critically ill patients.

[ref18] Curlin F. A, Nwodim C, Vance J. L, Chin M. H, Lantos J. D (2008). To Die, to Sleep: US Physicians’ religious and other objections to physician-assisted suicide, terminal sedation, and withdrawal of life support. American Journal of Hospice and Palliative Medicine.

[ref19] Donchin A (2000). Autonomy, interdependence and assisted suicide: Respecting boundaries/crossing lines Bioethics.

[ref20] Edelstein L, Temkin O, Temkin C. L (1967). Ancient medicine: Selected papers of Ludwig Edelstein.

[ref21] Emanuel E, Fairclough D, Daniels E, Clarridge B (1996). Euthanasia and physician-assisted suicide: Attitudes and experiences of oncology patients, oncologists, and the public. The Lancet.

[ref22] Emanuel E. J, Daniels E. R, Fairclough D. L, Clarridge B. R (1998). The practice of euthanasia and physician-assisted suicide in the United States. Journal of the American Medical Association.

[ref23] Emanuel E. J, Fairclough D. L, Emanuel L. L (2000). Attitudes and desires related to euthanasia and physician-assisted suicide among terminally ill patients and their caregivers. Journal of the American Medical Association.

[ref24] Finlay B (1985). Right to life versus the right to die: Some correlates of euthanasia attitudes. Sociologyand Social Research.

[ref25] Ferrand E, Dreyfus J, Chastrusse M, Ellien F, Lemaire F, Fischler M (2012). Evolution of requests to hasten death among patients managed by palliative care teams in France: A multicentre cross-sectional survey (DemandE). European Journal of Cancer.

[ref26] Foley K. M (1997). Competent care for the dying instead of physician-assisted suicide. New England Journal of Medicine.

[ref27] Fried T. R, Stein M. D, O’Sullivan P. S, Brock D. W, Novack D. H (1993). Limits of patient autonomy: Physician attitudes and practices regarding life-sustaining treatments and euthanasia. Archives of Internal Medicine.

[ref28] Ganzini L, Nelson H. D, Schmidt T. A, Kraemer D. F, Delorit M. A, Lee M. A (2000). Physicians’ experiences with the Oregon Death with Dignity Act. New England Journal of Medicine.

[ref29] Ganzini L, Block S (2002). Physician-assisted death —A last resort?. New England Journal of Medicine.

[ref30] Ganzini L, Dobscha S. K, Heintz R. T, Press N (2003). Oregon physicians’ perceptions of patients who request assisted suicide and their families. Journal of Palliative Medicine.

[ref31] Gaylin W, Kass L. R, Pellegrino E, Siegler M (1988). ‘Doctors must not kill’. Journal of the American Medical Association.

[ref32] Gendall P (2003). Massey survey shows support for euthanasia.

[ref33] General Medical Council (2006). Good Medical Practice.

[ref34] General Medical Council (2011). New guidance on dealing with complaints about assisting suicide.

[ref35] Georges J. J, The A. M, Onwuteaka-Philipsen B. D, van der Wal G (2008). Dealing with requests for euthanasia: A qualitative study investigating the experience of general practitioners. Journal of Medical Ethics.

[ref36] Gill M. B (2005). A moral defense of Oregon’s physician-assisted suicide law. Mortality.

[ref37] Gillon R, Downing A (1969). Suicide and voluntary euthanasia;historical perspectives. Euthanasia and the right to die.

[ref38] Goldney R. D (2012). Neither euthanasia nor suicide, but rather assisted death. Australian and New Zealand Journal of Psychiatry.

[ref39] Griffiths J, Weyers H, Adams M (2008). Euthanasia and the law in Europe.

[ref40] Haverkate I, van der Heide A, Onwuteaka-Philipsen B. D, van der Maas P. G, van der Wal J (2001). The emotional impact on physicians of hastening the death of a patient. Medical Journal of Australia.

[ref41] Health and Disability Commissioner (2009). Code of health and disability services consumers’ rights.

[ref42] Heath I (2012). What’s wrong with assisted dying?. British Medical Journal.

[ref43] Hendon H, Foley K (2008). Physician-assisted suicide in Oregon: A medical perspective. Michigan Law Review.

[ref44] Heyland D. K, Dodek P, Rocker G, Groll D, Gafni A, Pichora D, Lam M, for the Canadian Researchers End-of-Life Network(CARENET) (2006). What matters most in end-of-life care: Perceptions of seriously ill patients and their family members. Canadian Medical Association Journal.

[ref45] Hussain T, White P (2009). GPs’ views on the practice of physician-assisted suicide and their role in proposed UK legalisation: A qualitative study. British Journal of General Practice.

[ref46] Kass L, Kogan B. S (1991). Death with dignity and the sanctity of life. A time to be born and a time to die: The ethics of choice.

[ref47] Kass L. R (1998). Neither for love nor money: Why doctors must not kill. Public Interest.

[ref48] Kissane D. W, Clarke D. M, Street A. F (2001). Demoralization syndrome - a relevant psychiatric diagnosis for palliative care. Journal of Palliative Care.

[ref49] Kuhse H, Singer P, Baume P, Clark M, Rickard M (1997). End-of-life decisions in Australian medical practice. Medical Journal of Australia.

[ref50] Macleod S (2012). Assisted dying in liberalised jurisdictions and the role of psychiatry: A clinician’s view. Australian and New Zealand Journal of Psychiatry (online).

[ref51] Maltoni M, Pittureri C, Scarpi E, Piccinini L, Martini F, Turci P, Amadori D (2009). Palliative sedation therapy does not hasten death: Results from a prospective multicenter study. Annals of Oncology.

[ref52] Materstvedt L. J, Clark D, Ellershaw J, Forde R, Boeck Gravgaard A. M, Muller-Busch H. C (2003). Euthanasia and physician-assisted suicide: A view from an EAPC Ethics Task Force. Palliative Medicine.

[ref53] McCormack R, Clifford M, Conroy M (2012). Attitudes of UK doctors towards euthanasia and physician-assisted suicide: A systematic literature review. Palliative Medicine.

[ref54] Mehnert A, Vehling S, Höcker A, Lehmann C, Koch U (2011). Demoralization and depression in patients with advanced cancer: Validation of the German version of the demoralization scale. Journal of Pain and Symptom Management.

[ref55] Meier D. E, Emmons C. A, Wallenstein S, Quill T, Morrison R. S, Cassel C. K (1998). A national survey of physician-assisted suicide and euthanasia in the United States. New England Journal of Medicine.

[ref56] Michalsen A, Reinhart K (2006). “Euthanasia”: A confusing term, abused under the Nazi regime and misused in present end-of-life debate. Intensive Care Medicine.

[ref57] Mitchell C. B (1999-2000). Of euphemisms and euthanasia: The language games of the Nazi doctors and some implications for the modern euthanasia movement. Journal of Death and Dying.

[ref58] Mitchell K, Owens G (2004). End-of-life decision-making by New Zealand general practitioners: A national survey. New Zealand Medical Journal.

[ref59] Neil D. A, Coady C. A. J, Thompson J, Kuhse H (2007). End-of-life decisions in medical practice: A survey of doctors in Victoria (Australia). Journal of Medical Ethics.

[ref60] O’Reilly K. B (2010). Physician-assisted suicide legal in Montana, court rules. American Medical News: American Medical Association.

[ref61] Paris J. J (2009). Why involve physicians in assisted suicide?. The American Journal of Bioethics.

[ref62] Parks J. A (2000). Why gender matters to the euthanasia debate: On decisional capacity and the rejection of women’s death requests. The Hastings Center Report.

[ref63] Pellegrino E. D (1992). Doctors must not kill. Journal of Clinical Ethics.

[ref64] Pereira J (2011). Legalizing euthanasia or assisted suicide: The illusion of safeguards and controls. Current Oncology.

[ref65] Portenoy R. K, Coyle N, Kash K, Brescia F, Scanlon C, Doh J. D (1997). Determinants of the willingness to endorse assisted suicide: A survey of physicians, nurses, and social workers. Psychosomatics.

[ref66] Prichard J (2012). Euthanasia: A reply to Bartels and Otlowski. Journal of Law and Medicine.

[ref67] Putnam C (2009). What kind of a right is the ‘right to die’?. European Journal of Mental Health.

[ref68] Quill T. E (1991). Death and dignity. New England Journal of Medicine.

[ref69] Quill T. E, Battin M. P (2004). Physician-assisted dying: The case for palliative care and patient choice.

[ref70] Rachels J (1994). Active and passive euthanasia. New England Journal of Medicine.

[ref71] Rady M. Y, Verheijde J. L (2010). Continuous deep sedation until death: Palliation or physician-assisted death?. American Journal of Hospice and Palliative Medicine.

[ref72] Randall F, Downie R (2010). Assisted suicide and voluntary euthanasia: Role contradictions for physicians. Clinical Medicine.

[ref73] Remmel E (2011). Living while dying: Learning to live in the face of cancer. Psychology Today.

[ref74] Rosenblum V. G, Forsythe C. D (1990). The right to assisted suicide: Protection of autonomy or an open door to social killing. Issues in Law & Medicine.

[ref75] Salem T (1999). Physician-assisted suicide: Promoting autonomy or medicalizing suicide?. The Hastings Center Report.

[ref76] Seale C (2006). National survey of end-of-life decisions made by UK medical practitioners. Palliative Medicine.

[ref77] Seale C (2009). End-of-life decisions in the UK involving medical practitioners. Palliative Medicine.

[ref78] Seale C (2010). The role of doctors’ religious faith and ethnicity in taking ethically controversial decisions during end-of-life care. Journal of Medical Ethics.

[ref79] Sellman D (1995). Euphemisms for euthanasia. Nursing Ethics.

[ref80] Sessums L. L, Zembrzuska H, Jackson J. L (2011). Does this patient have medical decision-making capacity?. Journal of the American Medical Association.

[ref81] Silveira M. J, DiPiero A, Gerrity M. S, Feudtner C (2000). Patients’ knowledge of options at the end of life: Ignorance in the face of death. Journal of the American Medical Association.

[ref82] Singer P. A, Martin D. K, Kelner M (1999). Quality end-of-life care: Patients’ perspectives. Journal of the American Medical Association.

[ref83] Steele W. W, Hill B. B (1976). A plea for a legal right to die. Oklahoma Law Review.

[ref84] Steinhauser K. E, Christakis N. A, Clipp E. C, McNeilly M, McIntyre L, Tulsky J. A (2000). Factors considered important at the end of life by patients, family, physicians, and other care providers. Journal of the American Medical Association.

[ref85] Stolberg M (2007). Active euthanasia in pre-modern society 1500–1800: Learned debates and popular Practices. Social History of Medicine.

[ref86] Sullivan A. D, Hedberg K, Fleming D. W (2000). Legalized physician-assisted suicide in Oregon: The second year. New England Journal of Medicine.

[ref87] Tucker K. L, Steele F. B (2007). Patient choice at the end of life: Getting the language right. Journal of Legal Medicine.

[ref88] Vamos M. J (2012). Physician-assisted suicide: Saying what we mean and meaning what we say. Australian and New Zealand Journal of Psychiatry.

[ref89] van Delden J (1999). Slippery slopes in flat countries - a response. Journal of Medical Ethics.

[ref90] Van den Block L, Deschepper R, Bilsen J, Bossuyt N, Van Casteren V, Deliens L (2009). Euthanasia and other end of life decisions and care provided in final three months of life: Nationwide retrospective study in Belgium. British Medical Journal.

[ref91] van Marwijk H, Haverkate I, van Royen P, The A. M (2007). Impact of euthanasia on primary care physicians in the Netherlands. Palliative Medicine.

[ref92] Voluntary Euthanasia Society (2008). VESNZ survey shows that the majority of New Zealanders support medically assisted dying.

[ref93] Wanzer S. H, Federman D. D, Adelstein S. J, Cassel C. K, Cassem E. H, Cranford R. E, van Eys J (1989). The physician’s responsibility toward hopelessly ill patients. New England Journal of Medicine.

[ref94] Wilson D. M, Birch S, MacLeod R, Dhanji N, Osei-ware J, Cohen J (in press). The public’s viewpoint on the right to hastened death in Alberta: Findings from a population survey study. Health & Social Care in the Community.

[ref95] Wolf S, Wolf S. M (1996). Gender, feminism, and death: Physician-assisted suicide and euthanasia. Feminism & Bioethics. Beyond Reproduction.

